# Improving the Catalyst Efficiency for Hyperpolarization of Pyruvate Derivatives by Means of Hydrogenative PHIP

**DOI:** 10.1002/cmdc.202500379

**Published:** 2025-09-12

**Authors:** Ginevra Di Matteo, Oksana Bondar, Carla Carrera, Eleonora Cavallari, Sumit Mishra, Francesca Reineri

**Affiliations:** ^1^ Department Molecular Biotechnology and Health Sciences University of Torino Via Nizza 52 10126 Torino Italy; ^2^ National Research Council (CNR) Institute of Biostructures and Bioimages (IBB) Via Nizza 52 10126 Torino Italy

**Keywords:** hydrogenation, hyperpolarization, magnetic resonance imaging, metabolism, pyruvate

## Abstract

Hyperpolarized pyruvate is the most widely used probe for metabolic imaging in magnetic resonance (MR). Parahydrogen induced polarization‐ side arm hydrogenation allows to generate it through the catalytic hydrogenation of pyruvate esters. Due to the transient nature of MR hyperpolarization and to the fact that in vivo applications require a high amount of hyperpolarized substrate, a concentrated product solution must be obtained in a few seconds, therefore a high catalyst concentration is needed. The homogeneous rhodium catalyst used for the reaction can be deactivated by the hydrogenation products (or substrates) and the substrate‐to‐catalyst ratio becomes even lower, especially for pyruvate esters. The addition of tris‐phenyl phosphine to the hydrogenation mixture prevents the catalyst deactivation, when it is due to the hydrogenation product allyl pyruvate and the amount of catalyst needed to obtain a concentrated batch of hyperpolarized pyruvate ester has been reduced significantly. Following to hydrolysis and extraction of sodium pyruvate in aqueous phase, the concentration of the hyperpolarized metabolite has been increased to about 60 mM and ^13^C‐MRI experiments have been carried out using different dilution of the hyperpolarized metabolite in water.

## Graphical abstract

Hyperpolarization (HP) of pyruvate is obtained by means of the parahydrogen based method named parahydrogen induced polarization‐side arm hydrogenation. Cationic rhodium complexes are usually applied for the hydrogenation of the precursor, but the concentration needed to obtain complete reduction in a few seconds of a batch of substrate is relatively high. In this work, the amount of catalyst is reduced significantly while the concentration of HP metabolite is increased. 13C‐magnetic resonance imaging show that the molar polarization of the HP product is well sufficient for in vivo imaging studies.

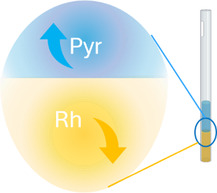

## 1 Introduction

The use of magnetic resonance imaging (MRI) in diagnostics provides anatomical and functional information about tissues, mainly exploiting the proton signal of water. A hidden potential is metabolic imaging, as the capability of nuclear magnetic resonance (NMR) to identify and quantify molecules noninvasively can provide a powerful tool for the investigation of metabolism in vivo. Nevertheless, these applications have been hampered by the intrinsic low sensitivity of MR‐based methods, that leads to time‐consuming acquisitions and low spatial resolution.

In the last two decades, hyperpolarization (HP) techniques and, in particular, dissolution‐dynamic nuclear polarization (d‐DNP) allowed to enhance the MR signals of some biologically relevant substrates by orders of magnitude, in the liquid state.^[^ ^1^, ^2^, ^3^ ^–^ ^4^ ^]^ This opened new perspectives to MR diagnostics as the investigation of metabolic pathways in vivo, in real time, became possible.^[^ ^2^ ^]^ Hyperpolarized [1‐13C]pyruvate has been widely exploited for the study of cancer and other pathologies in many preclinical studies and clinical trials,^[^ ^5^ ^]^ since it is at the crosspoint of different metabolic pathways.

Parahydrogen induced polarization (PHIP)^[^ 6, 7, 8, 9, 10, 11 ^]^ is a HP technique that can be easily handled in a chemistry laboratory, much more affordable and technically simpler than d‐DNP. Para and orthohydrogen are the two nuclear spin isomers of the H_2_ molecule,^[^ ^12^ ^,^ ^13^ ^]^ parahydrogen enrichment can be attained at cryogenic temperatures and exploited to generate high spin order on molecules by means of hydrogenative^[^ ^6^ ^,^ ^14^ ^,^ ^15^ ^]^ or nonhydrogenative‐PHIP.^[^ 16, 17, 18 ^]^ The former needs an unsaturated substrate (i.e., double or triple carbon–carbon bond) to which pairwise transfer of the two hydrogen atoms into magnetically different positions occurs. The resulting nuclear spin states populations are out‐of‐equilibrium and HP results.

**Scheme 1:**
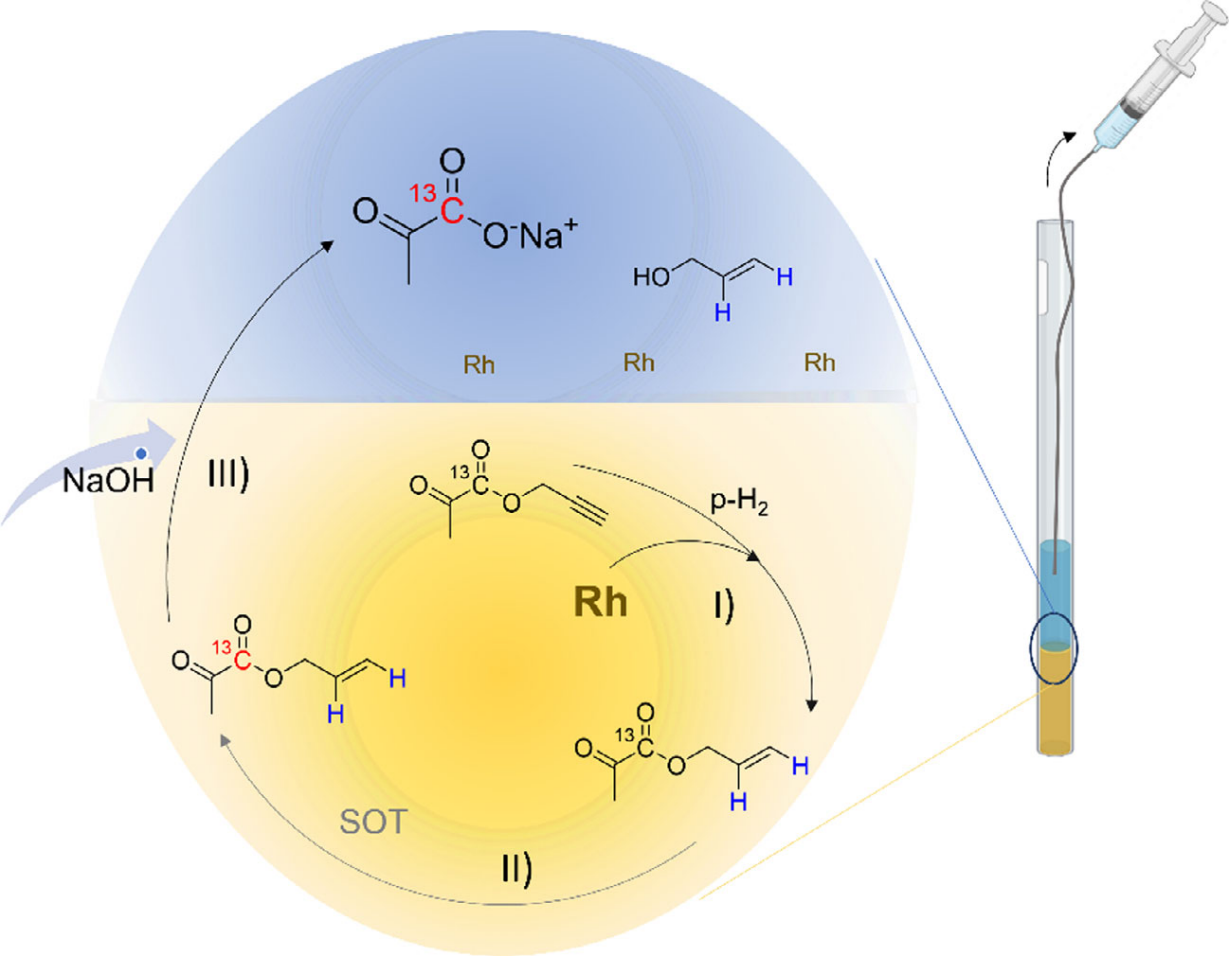
Schematic draw of the PHIP‐SAH method and phase transfer from the organic to the aqueous phase.

HP of pyruvate by means of hydrogenative PHIP is obtained through the side arm hydrogenation (PHIP‐SAH) method (**Scheme** **1**). ^[^ ^19^ ^–^ ^21^ ^]^ An ester derivative of pyruvate, bearing an unsaturation on the alcoholic moiety, is hydrogenated (step I), spin order is transferred (step II) from the parahydrogen protons to the carboxylate spin of pyruvate,^[^ 22, 23, 24, 25, 26 ^]^ the ester is hydrolyzed (step III), and the HP metabolite is obtained.

To be suitable for in vivo investigations, the molar polarization of the metabolic probe (i.e., the product between concentration and HP) must be sufficiently high.^[^ ^27^ ^]^ As a matter of reference, conventional ^1^H MRI relies on ^1^H‐MR signal of water that has, in a 9T magnetic field, molar polarization 3 mM (111 M ^1^H concentration at 0.003% polarization).^[^ ^28^ ^]^ Feasibility of ^13^C‐MRI is strongly limited by its concentration in vivo that, also in case of isotopically enriched metabolites, is in the submillimolar range.

As far as the PHIP‐SAH method is concerned, a concentrated batch of substrate (ester derivative of pyruvate) has to be hydrogenated completely (i.e. 100% yield) in a very short timescale, that is dictated by relaxation rate of the hyperpolarized signal and by the complex dynamics of hyperpolarized materials, especially at low magnetic field.^[^ ^29^ ^]^ Therefore the catalyst loading has to be high, compared to those usually applied in conventional organometallic reactions, that occurs in a minute or hour timescale.^[^ ^30^ ^]^

Biological studies, in cells and in vivo, require that an aqueous solution of the HP substrates is obtained, but organic solvents are usually preferred for hydrogenation catalysis and hydrophilic solvents, such as acetone,^[^ ^31^ ^]^ cannot be easily and quickly removed from an aqueous solution. When the reaction is carried out in a lipophilic solvent (e.g. chloroform), hydrolysis of the ester by means of an aqueous base leads to the separation of two phases where the HP molecule is extracted into water phase, from which the residual traces of solvent can be easily removed by filtration.^[^ ^32^ ^]^ The metal catalyst is also a major issue for in vivo studies, because homogeneous catalysts, the most efficient for h‐PHIP, cannot be removed by filtration. Phase extraction allows to retain the metal in the organic phase and its concentration is reduced to 30 μM.^[^ ^20^ ^]^

In this work, the hydrogenation of different SAH derivatives (vinyl and propargyl derivatives) of pyruvate is investigated, with the purpose of making the HP process less demanding in terms of metal catalyst while improving the molar polarization of the product. The concentration of metal in the aqueous phase has been reduced and the change of the catalyst efficiency with different substrates is reported.

## 2 Results and Discussion

Early investigations about PHIP^[^ ^33^ ^,^ ^34^ ^]^ demonstrated that, among the dihydride catalysts, cationic rhodium complexes containing a chelating phosphine [Rh(diene)diphos]+, (diphos: chelating phosphine; diene=cyclooctadiene or norbornadiene) give higher polarization level than other,^[^ ^35^ ^]^ thanks to the very short living reaction intermediates.^[^ ^36^ ^,^ ^37^ ^]^ Mixing between the nuclear spin states of hydrogen (*para* and *ortho* states) on transient species is minimized^[^ ^38^ ^]^ and spin order transfer to the product is improved, therefore these catalysts are usually preferred for h‐PHIP.

**Figure 1:**
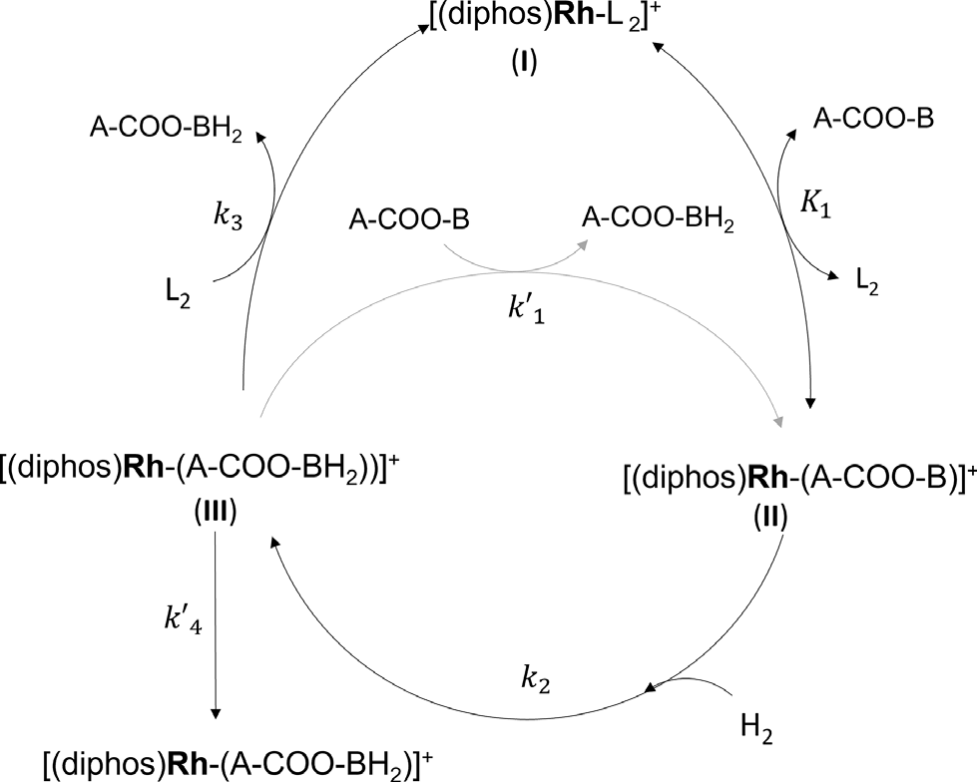
Catalytic pathway carried out by the cationic rhodium complexes having the general formula [Rh(diene)(diphos)]^+^[BF_4_]^−^(diphos: bis‐phosphine chelating ligand; diene: norbornadiene or cyclooctadiene). In the figure, L_2_ can represent either the diene, the solvent, or another ligand. The substrate is indicated with the generic formula A‐COO‐B, where A is the acidic moiety (either CH_3_ or COCH_3_ for acetate and pyruvate, respectively) and B the alcoholic moiety of the ester (either vinyl or propargyl alcohol). The light gray arrow indicates the alternative passage from intermediate III to II, instead of passages 1 and 3.

Mechanistic studies^[^ ^39^ ^,^ ^40^ ^]^ showed that the catalyst active form (I in **Figure** **1**) is generated through hydrogenation and displacement of the diene (norbornadiene or cyclooctadiene), that affords free coordination sites available for the incoming substrate, then the oxidative addition of hydrogen takes place in a passage that is rate‐determining (Figure 1).^[^ ^41^ ^,^ ^42^ ^]^

The catalyst efficiency for hydrogenation of vinyl and propargyl esters of acetate and pyruvate has been investigated in acetone and in chloroform. As already mentioned, polar solvents^[^ ^31^ ^]^ such as acetone or alcohols are usually preferred due to their capability to form solvated intermediates, while a lipophilic solvent (e.g. chloroform) allows the separation of the organic and aqueous phase after hydrolysis.

**Figure 2:**
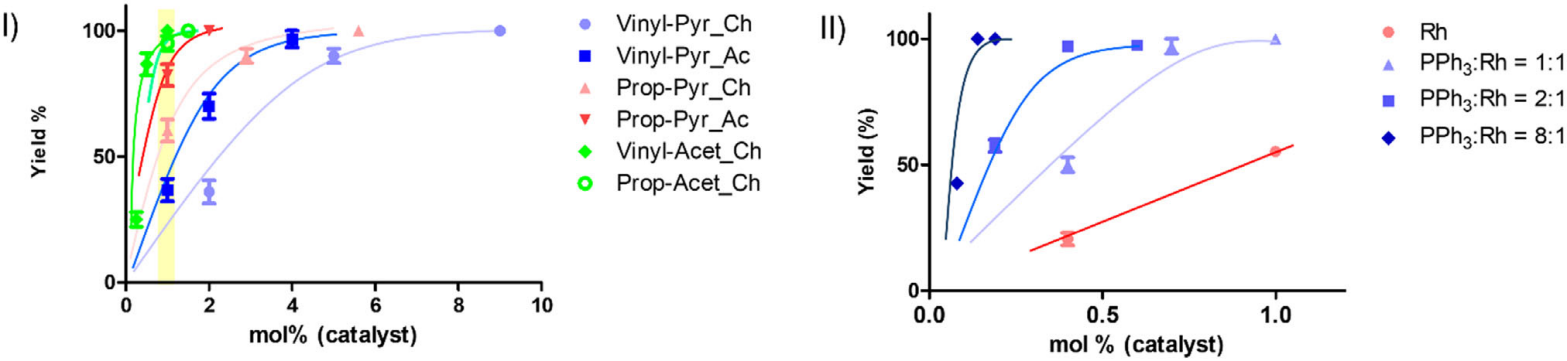
I) Hydrogenation yield reported as a function of the catalyst % ((catalyst/substrate)*100). Hydrogenation reactions are carried out at the same temperature (85 °C) and using the same hydrogen pressure (9 bar) in all the experiments. The yellow rectangle highlights the hydrogenation yield obtained using 1% catalyst with all the different substrates (Ac= acetone‐d_6_; Ch = chloroform‐d). II) Hydrogenation yield of propargyl‐pyruvate reported as a function of the catalyst %, with added triphenylphosphine (PPh_3_) at different ratios with respect to the catalyst. The experimental data are reported with the scattered plot and solid lines are just a guide for the eye. Experimental data are reported in the appendix, Tables S1 and S2, Supporting Information.

In order to avoid HP loss during hydrogenation, the reaction is completed in a few seconds, at high temperature (80–90 °C) and hydrogen pressure (9 bar) to maximize the reaction speed. Under these conditions, 100% hydrogenation of a solution (≈150 mM) of vinyl‐ to ethyl‐acetate or propargyl‐ to allyl‐acetate can be obtained in less than 10 s, using 1% catalyst (**Figure** **2**).

Conversely, the complete reduction, in a few seconds, of a concentrated batch of propargyl and vinyl pyruvate requires a higher catalyst percentage. In acetone, the catalyst increases to 2% and 4% for propargyl and vinyl pyruvate, respectively, while in chloroform about 4% and 8% catalyst is needed for the hydrogenation of the two esters (Figure 2). In other words, the substrate to catalyst ratio, that is 100:1 for the complete hydrogenation of a concentrated batch of vinyl acetate, decreases to 25:1 for propargyl‐pyruvate and almost 10:1 for vinyl‐pyruvate, in chloroform.

Further investigations showed that the reaction is stopped after 2–3 s, in chloroform, while in acetone slows down significantly, if carried out at 80 °C. (Figure S4I, Supporting Information). At lower temperature (60 °C), the catalyst efficiency is maintained for longer time in both solvents (Figure S4II, Supporting Information). These hydrogenation results demonstrate that the catalyst undergoes a fast deactivation process, especially at high temperature and in a poorly coordinating solvent, therefore the apparently low efficiency is due to subtraction of the active form from the hydrogenation mixture.

The hydrogenation time has a strong effect on the HP level,^[^ ^32^ ^,^ ^43^ ^]^ which becomes significantly lower (**Table** **1**), due to the fast decay of spin order, after that parahydrogen protons have been transferred to the product molecule. The difference between ^13^C polarization observed after 10 and 2 s hydrogenation is quite significant (30% less), therefore the hydrogenation has to be kept as fast as possible, and this implies the use of a high catalyst concentration.

**Table 1:** HP level obtained on the ^13^C carboxylate signal of allyl‐pyruvate (0.5 M, non‐^13^C labeled substrate), and sodium [1‐^13^C]pyruvate (≈0.05 M,^13^C labeled substrate). Different catalyst concentration and hydrogenation (shaking) times have been used. Hydrolysis of the allyl ester has been carried out using both a protonated and deuterated base, in the second case the HP loss on the metabolite is significantly lower than using NaOH in H_2_O.

Catalyst [%]a)	Solvent	Hydrogenation (s)	^13^C polarization [%] (allyl‐pyruvate)b)	^13^C polarization [%] (Na‐pyruvate)b)
4.5	CDCl_3_	2	7.4 ± 0.4%	
1.4	CDCl_3_	2	8.3 ± 0.5%	3.5 ± 0.5% NaOH + H_2_O 6.0 ± 0.5% (NaOD + D_2_O)
1.4	CDCl_3_	10	5.7 ± 0.3%	
1.4	Acetn‐d_6_	2	7.8 ± 0.8%	

a) Catalyst % is calculated as follows: ([catalyst]/[substrate])*100.

b) ^13^C polarization is calculated as reported in ref. [21].

Phosphines are widely employed in catalysis due to their ability to reversibly coordinate metal complexes and stabilize reaction intermediates, therefore the effect of triphenylphosphine (PPh_3_) on hydrogenation has been tested.^[^ ^42^ ^]^ The amount of PPh_3_ has been calculated considering the catalyst concentration and one, two, and eight equivalents have been added, in different experiments.

The hydrogenation efficiency is almost doubled by the addition of an equimolar amount of phosphine to rhodium and complete hydrogenation of propargyl to allyl pyruvate is obtained using 1% catalyst. Increasing the amount of PPh_3_, the complete reduction of a concentrated batch of pr propargyl pyruvate (0.5 m) is obtained in a few seconds using 1 mM catalyst in chloroform (0.2% catalyst) (Figure 2II).

Conversely, the hydrogenation of the two vinyl esters (vinyl acetate and pyruvate) is almost completely inactivated by the addition of PPh_3_ (Figure S5). The opposite effect of the phosphine on the hydrogenation of the two different ester derivatives can be explained considering the complete hydrogenation pathway and will be discussed more into details in the next paragraph.

Propargyl‐pyruvate has been preferred to the vinyl ester as a precursor of HP‐pyruvate, due to the higher hydrogenation efficiency. Following to hydrolysis, carried out using an aqueous base, the concentration of HP pyruvate, in the aqueous solution is 55 ± 5 mM. Although mixing between the aqueous and organic phase has to be improved and losses of organic and aqueous solution during hydrolysis have to be reduced, the molar polarization of pyruvate, calculated as the product between concentration and HP, is ≈2 mM when a protonated base is used for the hydrolysis and is almost doubled with NaOD in D_2_O.

It must be reminded that, in the first in vivo studies carried out using PHIP polarized [1‐13C] pyruvate, 1 mM polarization was reported at the timepoint of injection (i.e., 3.5% polarization^[^ ^44^ ^]^ and metabolite concentration about 35 mM). However, the molar polarization needed for in vivo ^13^C imaging studies depends on several factors, some of them are related to the MRI experiment, such as the type of imaging sequence (anatomic or chemical shift image), the target spatial resolution, and the time duration of the acquisition. Other are related to the in vivo experiment, for example the dilution of the HP agent in the circulation system. Some parameters such as the concentration of the HP agent, due to the tissue perfusion, and its relaxation speed in a specific region cannot be easily measured or predicted. Nevertheless, in order to get some clues about the applicability of the HP‐pyruvate solution herein reported, a few ^13^C‐MRI experiments have been carried out.

**Figure 3:**
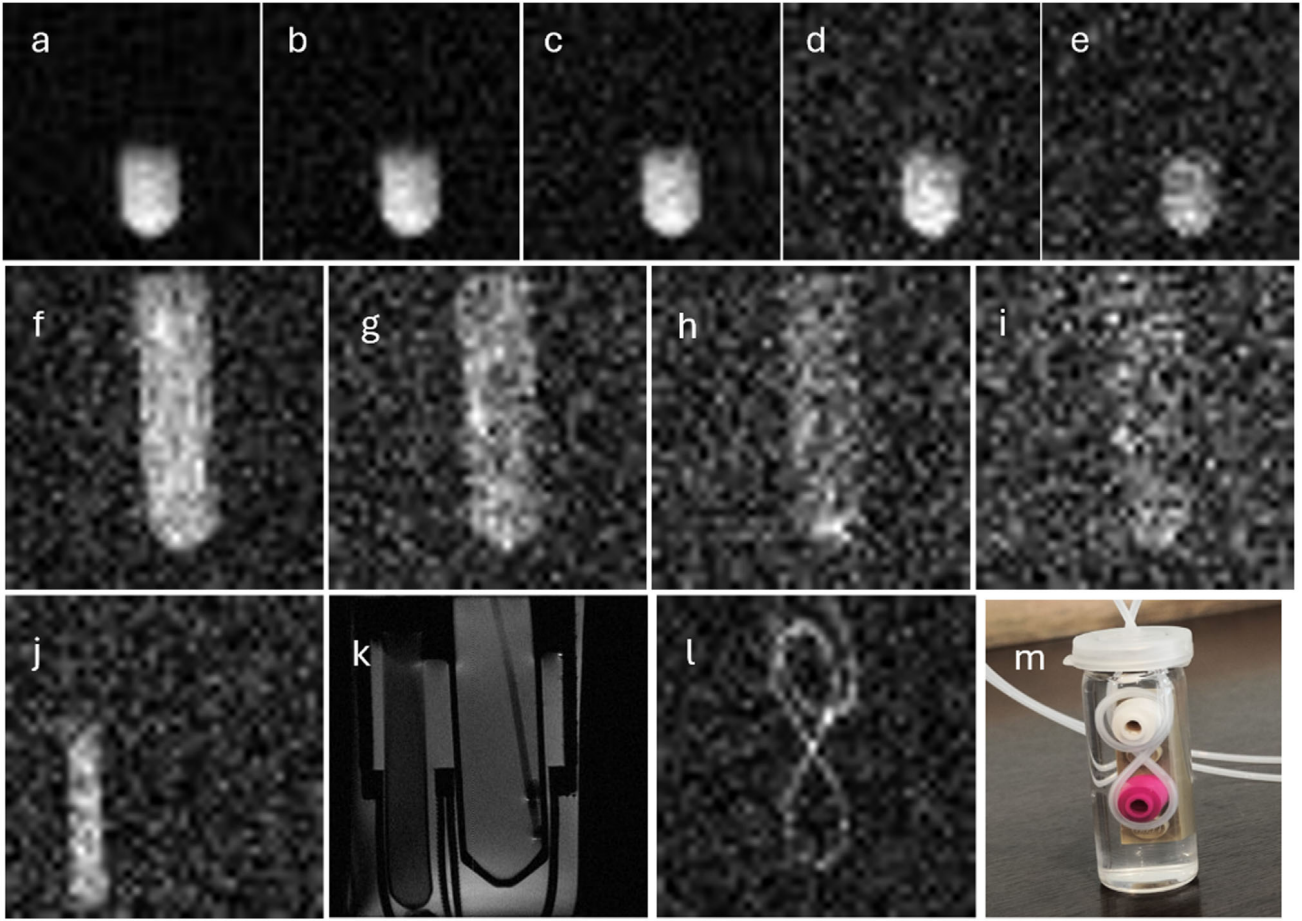
Single‐shot ^13^C‐ FLASH images have been acquired using the aqueous solution of HP‐pyruvate diluted in 1 ml H_2_O (images a to e; pyruvate concentration in the observed sample15 mM, molar polarization ≈0.5 mM) and 2 ml H_2_O (images f to I; pyruvate concentration in the observed sample 7–8 mM, molar polarization ≈0.25 M). Images a–e): 30° flip angle, FOV 36 × 36 mm, matrix size 32 × 32, TE 1.54ms; TR 3.31; scan time 106 ms. Images f–i): 30° flip angle, FOV 36 x 36 mm, matrix size 40 × 40, TE 1.54 ms; TR 3.31; scan time 140 ms. j) The ^13^C‐FLASH image of a thermally polarized urea (8 M, molar polarization 0.05 mM at 7T) gives the same SNR 5.6 after 16 averages (SNR in the first image HP is 7.5). k) ^1^H‐MR image of the phantom used for 13C‐MRI of the HP substrate (larger sample tube on the right) and 13C‐urea (8, 5 Mm sample tube on the left), the T_1_w RARE sequence has been acquired with the following parameters: TE = 20 ms, TR = 1000 ms, matrix size 256 × 256, FOV = 36 × 36mm; l) Single‐shot ^13^C‐ FLASH image (30° flip angle, FOV 36 × 36 mm, matrix size 40 × 40, TE 1.54 ms; TR 3.31; scan time 140 ms) of a capillary ETFE tube (i.d. 0.75 mm) filled with the aqueous solution of pyruvate (15 mM) obtained from hydrolysis using NaOD in D_2_O; m) photo of the phantom before being placed in the 7T MRI scanner for the injection of the HP solution through the capillary PTFE tube; to improve the homogeneity, the glass bottle has been filled with water, Lego bricks have been used to keep in place the ETFE tube.

The amount of aqueous solution (0.3 ± 0.05 ml) allows to obtain fast, time‐resolved imaging experiments using different dilution of the HP substrate (**Figure** **3**). The sequence of ^13^C‐FLASH images has been acquired using the aqueous solution of HP metabolite, after dilution in 1 ml and 2 ml H_2_O, which lead to a pyruvate concentration 15 and 8 mM, and a molar polarization 0.5 and 0.3 mM, respectively. A spatial resolution lower than 1 mm^3^ is obtained using HP‐pyruvate (15 mM in the image, polarization 0.9 mM) following to hydrolysis with the deuterated base.

The reduction of catalyst concentration leads also to a lower amount of metal in the aqueous solution. Rhodium is a slightly toxic heavy metal (single oral dose for rats LD_50_ 500–5000 mg Kg^−1^)^[^ ^45^ ^]^ and its concentration in the aqueous solution has been decreased from those reported (30  ×  10^−9^,^[^ ^20^ ^]^ 80 × 10^−9^^[^ ^46^ ^]^ to 2 × 10^−9 ^mol ml^−1^.

In order to have a reference for the molar polarization needed to obtain ^13^C‐MRI at a specific spatial resolution, a thermally polarized ^13^C Urea phantom (^13^C 8 M) has been used, corresponding to 0.05 mM polarization, at 7T. Using this sample, a fast imaging sequence (FLASH sequence, 30° excitation pulse) allows to obtain an in‐plane resolution 0.9 × 0.9 (slice thickness 5 mm) with 16 repetitions (Figure 3j, SNR ≈6).

### 2.1 Hydrogenation Kinetics

The changes of the hydrogenation kinetics observed with the different ester derivatives have been investigated further and the catalyst fate has been monitored by means of ^31^P‐NMR (Figure S1–S3, Supporting Information).

The hydrogenation of propargyl‐esters carried out at 20–25 °C, shows that the doublet (*δ *= 25 ppm, J_P‐Rh_ 143 Hz) of the catalyst precursor ([Rh(cod)(dppb][BF_4_]), given by the two chemically equivalent phosphorus atoms, disappears and other specimens are obtained, in which the two phosphorus atoms are placed in chemically different sites (Figure S1 and S2, Supporting Information). If the catalyst degradation, i.e. the decay of the ^31^P doublet, is reported as a function of allyl‐pyruvate percentage, the deactivation process is faster in chloroform than in acetone, and in the hydrogenation of propargyl‐acetate, it is even slower than for the pyruvate ester derivative (Figure S3, Supporting Information). The slower catalyst deactivation is consistent with the higher efficiency in the hydrogenation of the acetate derivative. It is well known that catalyst deactivation at high substrate‐to‐catalyst ratios is due to substrate inhibition^[^ ^47^ ^]^ and the stable rhodium complexes observed in the ^31^P spectra are likely given by the coordination of the hydrogenation products to the metal.

**Scheme 2:**
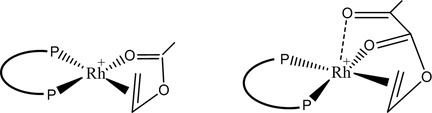
Coordination of vinyl acetate (left) and vinyl pyruvate (right) to the rhodium complex.

By monitoring the hydrogenation of vinyl esters through ^31^P spectra, we observe that, for vinyl acetate, the active form of the catalyst (i.e. the ^31^P signal of the two chemically equivalent atoms) is maintained until its complete reduction to ethyl ester. Conversely, using vinyl pyruvate, the catalyst deactivation occurs without that any ethyl‐pyruvate is obtained. At low temperature and hydrogen pressure vinyl‐pyruvate forms an unactive adduct with the catalyst and the hydrogenation reaction cannot proceed. This is consistent with the low catalyst efficiency in the hydrogenation of vinyl‐pyruvate, when the reaction is carried out at high temperature and high pressure. This can be justified considering that the pyruvate derivative has three coordination sites to the rhodium center (**Scheme** **2**) and can form an inactive addict, as reported for other substrates.^[^ ^48^ ^]^

The effect of the different ligands (solvent, hydrogenation product, substrate, or phosphine), on the hydrogenation kinetics can be rationalized considering the rate law describing the hydrogenation pathway, reported by Halpern (Equation 5) (Figure 1).^[^ ^42^ ^]^ The exchange process (1) and the rate determining step (2) are taken into account

(1) $${\left[\mathrm{Rh}\right]}^{+}+A-\mathrm{COO}-B\;\overset{K_{1}}{↔}\;{\left[\mathrm{Rh}\left(A-\mathrm{COO}-B\right)\right]}^{+}$$

(2) $${\left[\mathrm{Rh}\left(A-\mathrm{COO}-B\right)\right]}^{+}+H_{2}\;\overset{k_{2}}{\to }\;{\left[A-\mathrm{COO}-{\mathrm{BH}}_{2}\right]}^{+}$$

where $K_{1}$ is an equilibrium constant ($K_{1}=\frac{k_{1}^{\mathrm{dir}}}{k_{1}^{\mathrm{inv}}}$)

The concentration of intermediate II is supposed stationary during the reaction (3) and, being the total amount of rhodium fixed (4)

(3) $$\frac{d\left[\mathrm{Rh}\left(\text{ACOOB}\right)\right]}{\mathrm{dt}}=0$$

is

(4) $${\left[\mathrm{Rh}\right]}_{\mathrm{tot}}=\left[{\mathrm{Rh}}^{+}\right]+\left[\mathrm{Rh}\left(\text{ACOOB}\right)\right]$$

the rate law is derived is

(5) $$\frac{d\left[\text{ACOOB}H_{2}\right]}{\mathrm{dt}}=\frac{k_{2}K_{1}\left[H_{2}\right]\left[\text{ACOOB}\right]{\left[\mathrm{Rh}\right]}_{\mathrm{tot}}}{1+K_{1}\left[\text{ACOOB}\right]}$$

assuming that $k_{1}^{\mathrm{inv}}\gg k_{2}[H_{2}]$.

In that equation, the ligand L_2_ and the intermediate III do not appear, while these have to be taken into account in the experiments herein reported. To do this, the kinetic equation has been rewritten considering the time‐dependent concentration of intermediates II and III as follows

(6) $$\frac{d\left[\mathrm{Rh}\left(\text{ACOOB}\right)\right]}{\mathrm{dt}}=k_{1}^{\mathrm{dir}}\left[\text{ACOOB}\right]\left[RhL_{2}\right]-k_{1}^{\mathrm{inv}}\left[L_{2}\right]\left[\mathrm{Rh}\left(\text{ACOOB}\right)\right]-k_{2}\left[\mathrm{Rh}\left(\text{ACOOB}\right)\right]\left[H_{2}\right]$$

(7) $$\frac{d\left[\mathrm{Rh}\left(\text{ACOOB}H_{2}\right)\right]}{\mathrm{dt}}=k_{2}\left[\mathrm{Rh}\left(\text{ACOOB}\right)\right]\left[H_{2}\right]-k_{3}\left[\mathrm{Rh}\left(\text{ACOOB}H_{2}\right)\right]\left[L_{2}\right]$$

and the rate of the reaction

(8) $$\frac{d\left[\text{ACOOB}H_{2}\right]}{\mathrm{dt}}=k_{3}\left[\mathrm{Rh}\left(\text{ACOOB}H_{2}\right)\right]\left[L_{2}\right]$$

Assuming, as in the original model, that the concentration of the two intermediates is stationary and that the catalyst amount is fixed

(9) $${\left[\mathrm{Rh}\right]}_{\mathrm{tot}}=\left[{\mathrm{RhL}}_{2}\right]+\left[\mathrm{Rh}\left(\text{ACOOB}\right)\right]+\left[\mathrm{Rh}\left(\text{ACOOB}H_{2}\right)\right]$$

It can be found that the speed of the hydrogenation reaction becomes

(10) $$\frac{d\left[\text{ACOOB}H_{2}\right]}{dt}=\frac{k_{2}\left[H_{2}\right]\left[\text{ACOOB}\right]{\left[Rh\right]}_{tot}}{\frac{\left(k_{1}^{\mathrm{inv}}\left[L_{2}\right]+k_{2}\left[H_{2}\right]\right)}{k_{1}^{\mathrm{dir}}}+\left(1+\frac{k_{2}\left[H_{2}\right]}{k_{3}\left[L_{2}\right]}\right)\left[\text{ACOOB}\right]}$$

This more general model can be reduced to the original one (Equation 5) when $k_{3}\approx k_{1}^{\mathrm{inv}}$ and $k_{1}^{\mathrm{inv}}[L_{2}]\gg k_{2}[H_{2}].$

It must be noticed that the assumption $k_{3}\approx k_{1}^{\mathrm{inv}}$ is valid only if the coordination strength of the stabilizing ligand (L_2_) is higher than that of both substrate and hydrogenation product. This occurs in the hydrogenation of vinyl acetate, where the hydrogenation product (ethyl acetate) has low coordination energy. Conversely the allyl esters form stable adducts with the metal, as observed using the ^31^P‐NMR. Their coordination strength is higher, especially for allyl pyruvate, and, also in acetone, $k_{3}[L_{2}]\ll k_{1}^{\mathrm{inv}}[L_{2}]$.

In this case, and considering that $k_{1}^{\mathrm{inv}}[L_{2}]\gg k_{2}[H_{2}]$, (Equation 10) becomes

(11) $$\frac{d\left[\text{ACOOB}H_{2}\right]}{dt}=\frac{k_{2}\left[H_{2}\right]K_{1}\left[\text{ACOOB}\right]{\left[Rh\right]}_{tot}}{1+K_{1}\left[\text{ACOOB}\right]+K_{1}\frac{k_{2}\left[H_{2}\right]}{k_{3}\left[L_{2}\right]}\left[\text{ACOOB}\right]}$$

in which it is clear that low $k_{3}[L_{2}]$ will bring to a decrease of the reaction rate, as observed in the hydrogenation of propargyl pyruvate.

The addition of the PPh_3_ ligand increases $k_{3}[L_{2}]$ and, being $k_{3}[L_{2}]\approx k_{1}^{\mathrm{inv}}\gg k_{2}[H_{2}]$, (Equation 5) is obtained. This occurs when the phosphine is added to the hydrogenation of propargyl pyruvate in chloroform or acetone.

When the ligand coordination strength is higher than the substrate, $k_{3}[L_{2}]\approx k_{1}^{\mathrm{inv}}\gg k_{1}^{\mathrm{dir}}[L_{2}]$, the equilibrium constant ( $K_{1}=\frac{k_{1}^{\mathrm{dir}}}{k_{1}^{\mathrm{inv}}}$) decreases and the reaction becomes slower. This is the situation observed when the phosphine is added to the hydrogenation of the vinyl esters.

The effect of the phosphine on the efficiency of the hydrogenation catalyst has been explained considering the entire hydrogenation mechanism. Being involved in the stabilization of intermediate I, it shifts the equilibrium (first step of the hydrogenation mechanism) and also the last step of the reaction (i.e. the product displacement from the metal center). The phosphine has a protecting effect against the allyl esters (especially allyl pyruvate), derived from hydrogenation of the propargylic derivatives. Conversely, it has an inhibiting effect on the hydrogenation of vinyl‐esters because the first step of the reaction, i.e. substrate exchange on the metal complex, is shifted. The hydrogenation process described by Equation (10) allows to take into account the different effects of solvent, hydrogenation product, and ligands on the kinetics of the reaction.

## 3 Conclusion

It has been shown that the changes of the relative stability of the reaction intermediates, given by different substrates, can lead to opposite effects on the reaction kinetics. In other words, the hydrogenation speed can change dramatically due to even small changes of the substrate.

Using the herein reported ester derivatives, it has been observed that the apparently different catalyst efficiency is due to the formation of unactive adducts of the metal complex with the hydrogenation product (in particular with allyl‐pyruvate) or the substrate itself (vinyl‐pyruvate). The coordination capacity of pyruvate derivatives on the rhodium center makes these substrates more demanding, in terms of catalyst percentage, than those of acetate. The addition of a few equivalents of PPh_3_ to the catalyst allows to prevent its inactivation given by the coordination of allyl‐pyruvate to the rhodium center, therefore the catalyst needed to obtain the complete reduction, in a few seconds, of triple to double bond is significantly reduced. Conversely, the use of vinyl‐pyruvate as a precursor, catalyst inactivation occurs through the coordination of the substrate to the metal. The addition of PPh_3_, that competes with the substrate for the coordination to the metal, leads to further inhibition of the hydrogenation reaction, observed with both pyruvate and acetate esters.

It has also been observed that, without the addition of PPh_3_, the catalyst degradation is faster in a noncoordinating solvent such as chloroform, therefore the needed percentage of metal complex in higher than in acetone. Nevertheless, the lipophilic solvent allows to remove the hydrogenation solvent quickly, while the use of acetone does not show any advantage from the point of view of HP level. In the end, the addition of the adjuvant ligand allows to reduce the rhodium concentration in chloroform almost ten times.

The concentrated batch of a HP pyruvate ester leads to an increase of the concentration of the HP metabolite in the aqueous solution to 55 ± 5 mM and a ^13^C polarization 3.5 ± 0.5% when hydrolysis is carried out using sodium hydroxide in H_2_O and 6.0 ± 0.5% if the deuterated base is used.

The imaging experiments, carried out using the aqueous solution of HP pyruvate, show that the molar polarization obtained using the herein reported procedure is well sufficient for imaging studies in vivo, while the biocompatibility of the agent solution has been improved significantly with respect to that previously reported thanks to the decrease of rhodium concentration to 2 μM.

## 4 Experimental Section

4.1

4.1.1

##### General Information

The catalyst ([1,4‐bis(diphenylphosphino)butane](1,5‐cyclooctadiene)rhodium(I) tetrafluoroborate] and vinyl acetate was purchased form Sigma‐Aldrich and used without further purification. Deuterated solvents were bought from CortecNet and used without purification. The propargylic and vinyl esters of [1‐13C]pyruvate and acetate were synthesized as reported previously.^[^ ^21^ ^]^

NMR spectra were measured using a Bruker Avance 600 MHz spectrometer. 92% *para*‐enriched hydrogen was produced using Bruker ParaHydrogen Generator (BPHG). Magnetic field cycling was carried out using the experimental setup reported previously,^[^ ^32^ ^]^ in brief, made of a triple‐layer mu‐metal shield equipped with a solenoid coil fed with electric current provided by an Arbitrary Waveform Generator (Agilent 33220A AWG). ^13^C polarization level was determined according to the reported procedure.^[^ ^49^ ^]^

MR images were acquired using a Bruker 7T Avance NEO 300 MRI scanner (Bruker Biospin, Ettlingen, Germany) equipped with a 30 mm ^1^H‐^13^C TR coil. ^13^C FLASH images were acquired with the following parameters: FA = 30° f, FOV 36 × 36 mm, matrix size 32 × 32 (Figure 3a–e) and 40 × 40 (Figure 3f–i) TE = 1.54 ms; TR = 3.31, single average, number of slices 1, slice thickness 5 mm.

##### Hydrogenation Reactions

NMR sample tubes equipped with a gas‐tight valve (Norell NMR sample tubes) were used to carry out the hydrogenation reactions.

In order to test the catalyst efficiency in chloroform and acetone, using the different substrates, the following solutions have been prepared, charged in the NMR tubes and immediately frozen in liquid nitrogen. At least three replicates have been carried out for each condition.

Vinyl pyruvate in chloroform‐d: 19 μmol substrate (final concentration 125 mM) and 1.7, 0.79 or 0.4 μmol catalyst (final concentration 11, 6 and 3 mM) in 150 μL chloroform‐d (corresponding to a catalyst percentage, with respect to the substrate, 9, 5 and 2%).

Vinyl pyruvate in acetone‐d_6_: 25.5 μmol (final concentration 170 mM) and 1.0, 0.55, or 0.27 μmol catalyst (final concentration 6.7, 3.4, and 1.8 mM) in 150 μL acetone‐d_6_ (corresponding to catalyst percentage, with respect to the substrate, 4, 2 and 1%).

Propargyl pyruvate in chloroform‐d: 25 μmol substrate (final concentration 170 mM) and 1.4, 0.7, or 0.3 μmol catalyst (final concentration 9, 5, 2 mM) in 150 μL chloroform‐d (corresponding to a catalyst percentage, with respect to the substrate, 5.6, 3, 1.2%).

Propargyl pyruvate in acetone‐d_6_: 25 μmol substrate (final concentration 170 mM) and 0.55 or 0.3μmol catalyst (final concentration 3.7 or 2 mM) in 150 μL acetone‐d_6_ (corresponding to a catalyst percentage, with respect to the substrate, 2.2 and 1.2%).

Vinyl acetate: 25 μmol substrate (final concentration 170 mM) and 0.3, 0.12, and 0.06 μmol catalyst (final concentration 2, 0.8, and 0.4 mM) in 150 μL chloroform‐d (corresponding to a catalyst percentage, with respect to the substrate, 1, 0.5, and 0.25%).

Propargyl acetate: 25 μmol substrate (final concentration 170 mM) and 0.4 or 0.25 μmol catalyst (final concentration 2.5 or 2 mM) in 150 μL chloroform‐d (corresponding to a catalyst percentage, with respect to the substrate, 1.5 and 1%).

The addition of PPh_3_ to the catalyst has been tested using the following solutions:

(Catalyst‐PPh_3_ = 1:1): 0.25, 0.15, or 0.1 μmol catalyst, 1 equivalent PPh_3_ and 25 μmol propargyl pyruvate (final concentration 170 mM) were dissolved in 150 μL chloroform‐d. Catalyst percentage, with respect to the substrate, is 1, 0.6, and 0.4%.

(Catalyst‐PPh_3_ = 1:1): 0.3 μmol catalyst, 1 equivalent PPh_3_, and 25 μmol vinyl acetate (final concentration 170 mM) were dissolved in 150 μL chloroform‐d. Catalyst percentage, with respect to the substrate, is 1.1%.

(Catalyst‐PPh3 = 1:2): 0.3, 0.2, or 0.1 μmol catalyst, 2 equivalents PPh_3_, and 50 μmol propargyl pyruvate (final concentration 330 mM) were dissolved in 150 μL chloroform‐d. Catalyst percentage, with respect to the substrate, is 0.6, 0.4, and 0.2%.

(Catalyst‐PPh3 = 1:8): 0.1, 0.075, or 0.05 μmol catalyst with 8 equivalents PPh_3_ and 50 μmol propargyl pyruvate (final concentration 330 mM) were dissolved in 150 μL chloroform‐d. Catalyst percentage, with respect to the substrate, is 0.2, 0.15, and 0.1%.

The solutions have been prepared, immediately charged in the NMR tubes, and frozen in liquid nitrogen.

The frozen tube was connected to a vacuum line and air was removed by applying vacuum (<0.2 mbar). Hydrogen (2.5 bar) was added keeping the NMR tube in a liquid nitrogen bath. For simplicity, parahydrogen obtained from BPHG Bruker ParaHydrogen Generator and stored in a 1 L cylinder has been used. After hydrogen addition, the NMR tube was disconnected from the vacuum line and kept frozen in liquid nitrogen (77 K) in order to prevent any chemical reaction, until the start of hydrogenation.

To carry out the hydrogenation reactions, the NMR tube containing the frozen reaction mixture was heated in a hot water bath (80 °C or 60 °C for 5 s), then shaken vigorously for different time delays as specified in Tables S2 and S3, Supporting Information. Immediately after shaking, the sample tube was opened to release hydrogen pressure and stop the hydrogenation reaction. 200 μl of solvent (either chloroform or acetone‐d6) were added, to obtain a sufficient volume for the NMR acquisition. Hydrogenation yield was determined from integration of the ^1^H‐NMR signals of the product and the substrate.

At least three replicates have been carried out for each condition.

##### Hyperpolarization Experiments

The samples for the HP experiments were prepared by adding 80 mmol propargyl‐pyruvate to a catalyst solution made with 3 μmol catalyst in 150 μL chloroform‐d (high catalyst concentration) or to a solution made with 0.3 μmol catalyst plus 2.4 μmol PPh_3_, dissolved in 150 μL chloroform‐d (high catalyst concentration), or to a solution made with 0.3 μmol catalyst plus 2.4 μmol PPh_3_, dissolved in 150 μL chloroform‐d (low catalyst load).

The solution was put into the NMR tube and immediately frozen, parahydrogen addition and hydrogenation reaction were carried out as reported before for the hydrogenation experiments.

The spin order transfer from the parahydrogen protons to ^13^C of the carboxylate group was obtained by applying magnetic field cycle, as reported previously.^[^ ^32^ ^]^ The magnetic field profile consists, in these experiments, in a diabatic step from 1.5 μT (starting magnetic field) to 50 nT (<1 ms) and an adiabatic re‐magnetization (linear) to 10 μT in 4 s.

The sample tube was removed from the shield, 250 μl of solvent were added, and the NMR tube was placed into the NMR spectrometer where the ^13^C‐NMR spectrum (one shot, 90° flip angle, RG 9) was acquired. The HP level was determined as reported in ref. [21]

In case that the aqueous solution of HP metabolite has to be obtained, the NMR tube containing the HP ester went to the hydrolysis step, instead of going to the NMR spectrometer for the acquisition.

##### Hydrolysis

Hydrolysis of allyl‐ester of pyruvate was carried out using an aqueous base solution (NaOH 0.13 N) containing sodium ascorbate (50 mM). For each experiment, 320 μl of this solution were charged into a PTFE tube (PTFE 1/16” × 0.75 i.d. tube, VICI Jour). The tube was pressurized with Argon (1.5 bar) and heated in a hot water bath (80 °C), as reported previously.^[^ ^32^ ^]^ In order to obtain the hydrolysis of the HP allyl‐ester of pyruvate, the NMR tube containing the solution of the HP product was open immediately after the end of the spin order transfer step (MFC) and the heated aqueous base was injected through the organic solution.

The separation between the organic and the aqueous phase occurs in a few seconds (5–8 s), then an acidic buffer (HEPES 144 mM, 100 μl, pH 5.4) was added to obtain neutral pH.

In the end, the aqueous phase was collected in a syringe, diluted with 0.3 ml D_2_O and injected, through a PTFE tube, into the test tube containing 0.5 or 1.5 ml H_2_O for the acquisition of the HP‐^13^C images.

The concentration of [1‐^13^C]pyruvate in the imaging phantoms has been measured by means of ^1^H NMR spectra using 3‐(trimethylsilyl)propionic‐2,2,3,3‐d_4_ acid sodium salt an internal reference.

##### Rhodium ICP Measurement

Rhodium was quantified by means of inductively coupled plasma mass spectrometry (ICP‐MS Element‐2; Thermo‐Finnigan, Rodano (MI), Italy). An aliquot of 0.2 ml of the aqueous solution of pyruvate obtained after hydrolysis of the allyl ester and phase extraction was mixed with 2.2 ml of HNO_3_ (70%) and introduced in the microwave digestion system. The following heating protocol was used: ramp up to 150 °C in 8 min, followed by 6 min at 150 °C. After mineralization the samples were collected and diluted with an appropriate volume of HNO_3_ (1%) in ultrapure water. Calibration curves were obtained using standard metal solutions (Sigma–Aldrich) in the range 0.1–0.005 μg ml^−1^.

## Conflicts of Interest

The authors declare no conflict of interest.

## Supporting information

Supplementary Material

## Data Availability

The data that support the findings of this study are available from the corresponding author upon reasonable request.
