# Supplementary material for: Improving the Catalyst Efficiency for Hyperpolarization of Pyruvate Derivatives by Means of Hydrogenative PHIP
Source: ChemMedChem. 2025 Sep 12;20(19):e202500379. doi: 10.1002/cmdc.202500379 (PMC12503905; doi:10.1002/cmdc.202500379)
Supplement: Supplementary file 1 — Supplementary Material [file CMDC-20-e202500379-s001.pdf]

Supporting information to

Improving the catalysts efficiency for the hyperpolarization of pyruvate derivatives by means of hydrogenative-PHIP.

Ginevra Di Matte202500379o, Oksana Bondar, Carla Carrera, Eleonora Cavallari, Sumit Mishra, Francesca Reineri

^{31}P-NMR

Hydrogenation of propargyl pyruvate and acetate have been followed by means of ^{31}P-NMR. In these experiments, the reactions have been carried out as reported in the main manuscript, shaking the NMR tube at 25°C (instead of 80°C) and using 4 bar hydrogen (instead of 8-9).

The NMR tubes have been charged with 150 μmol substrate (final concentration 450mM) and 2.7μmol catalyst (final concentration 8 mM) in 350 μL chloroform-d or acetone-d_6.

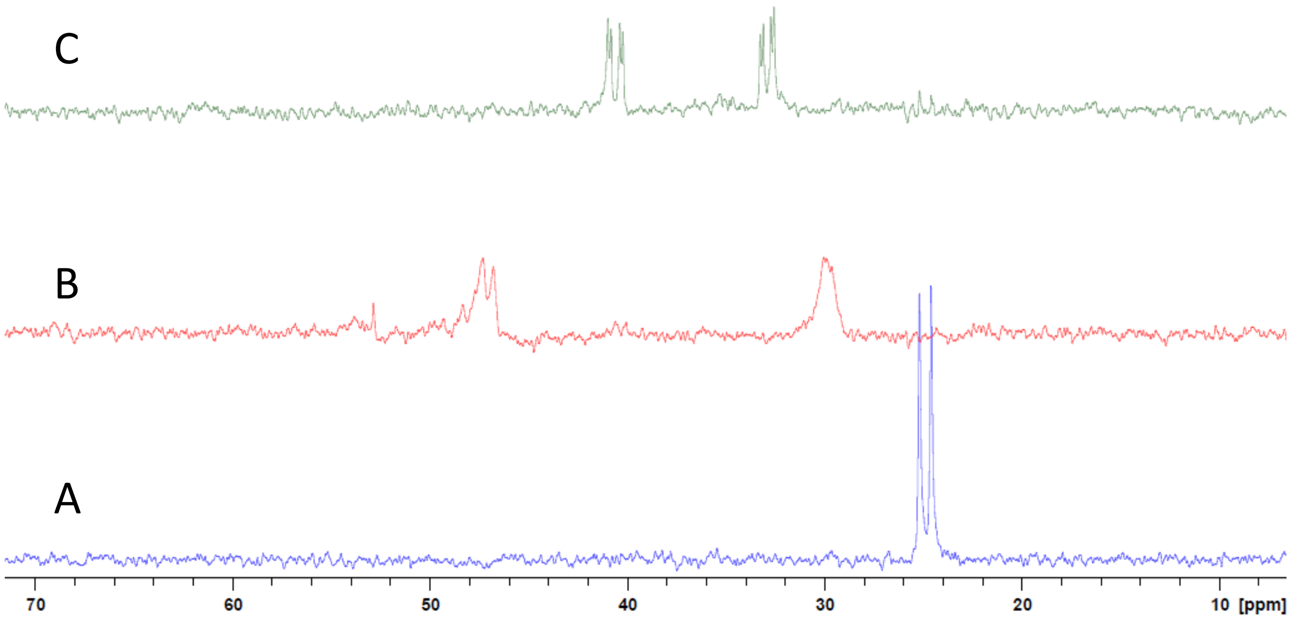

Figure S1. ^{31}P-NMR spectra of [Rh(COD)dppb]BF4 before (A) and after hydrogenation of propargyl-pyruvate (B) and propargyl-acetate (C) in chloroform.

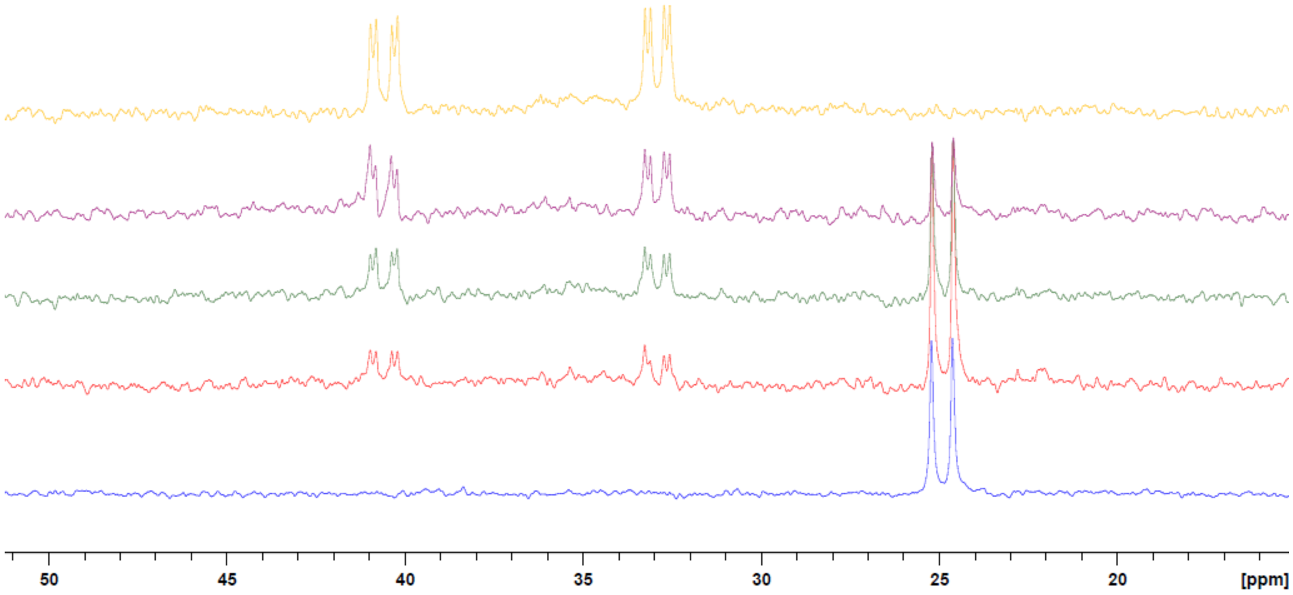

Figure S2. ^{31}P-NMR spectra during hydrogenation propargyl to allyl acetate. The signal of the active form of the catalyst (25ppm) disappears and other two doublets are formed.

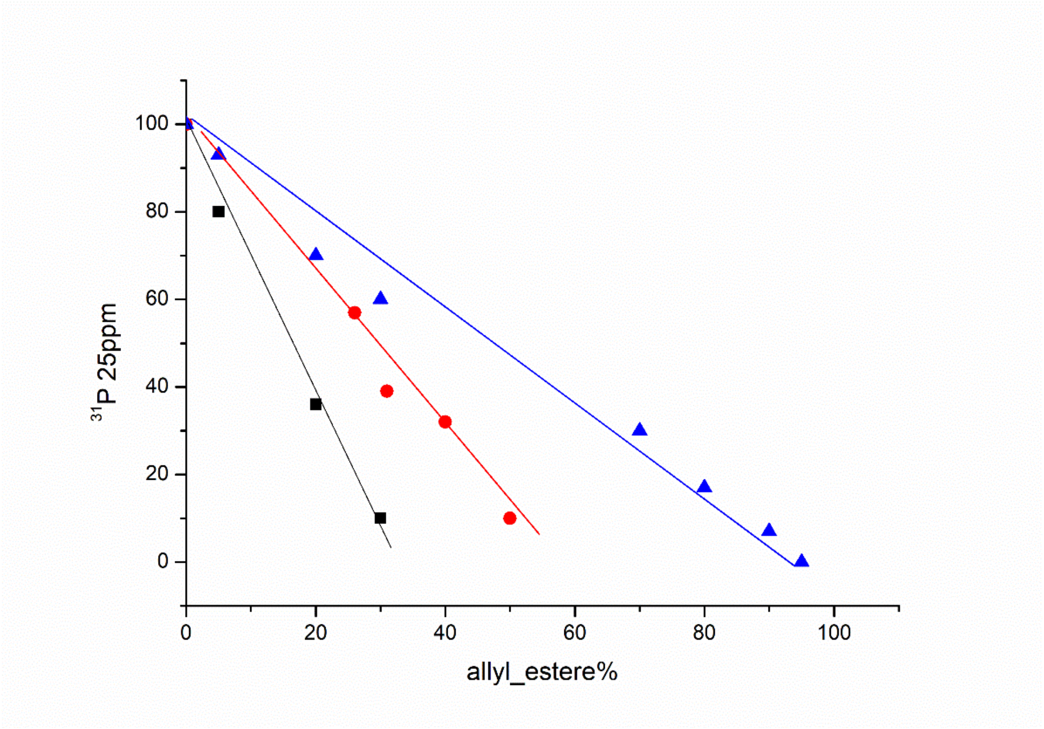

Figure S3. The intensity of the 31P signal at 25 ppm (active form of the catalyst) is reported as a function of the percentage of allyl ester produced from hydrogenation of: propargyl-acetate in chloroform (blue triangles); propargyl pyruvate in acetone (red dots) and propargyl pyruvate in chloroform (black squares).

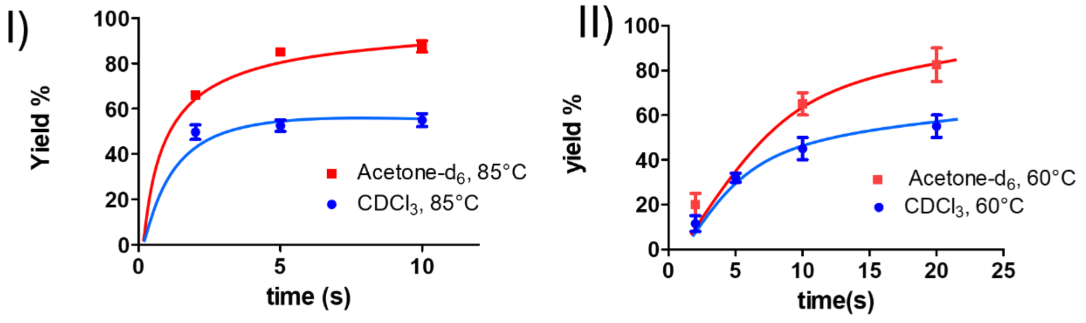

Figure S4. Hydrogenation yield at 85°C (I) and 60°C (II) for propargyl-pyruvate, using 1% catalyst, for different hydrogenation time (2, 5 and 10 seconds at 80°C, 2, 5, 10 and 20 seconds at 60°C).

solvent	Hydrogenation time	Temp	yield
$CDCl_3$	10”	85°C	55±2
	5”		53±2
	2”		50±2
Acetn	10	85°C	85±3
	5”		80±3
	2”		65±5

Table ST3. Data reported in figure S4.I (all the reactions have been carried out using 1% catalyst)

solvent	Hydrog. time	Temp	yield
$CDCl_3$	20”	60°	55±5
	10”		50±5
	5”		30±5
	2”		15±2
Acetn	20”	60°	80±5
	10”		65±2
	2”		20±2

Table ST4. Data reported in figure S4.II (all the reactions have been carried out using 1% catalyst)

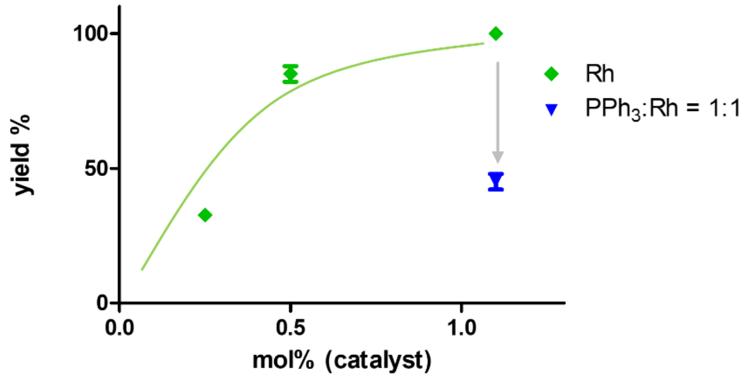

Figure S5. Hydrogenation yield of vinyl acetate (I) is reported as a function of the catalyst %, with triphenylphosphine (PPh_3) added, in different ratios to the catalyst. The arrow evidences the phosphine inhibition effect, in the hydrogenation of the vinyl-ester. Experimental data are reported in table ST5.

Cat%	PPh3 equivalents	yield
1.1%		100
0.6%		95±5
0.3%		33±5
1.1	1	45±5

Table ST5. Data reported in figure S.5

Data tables

substrate	solvent	Cat%	yield
Vinyl-pyruvate	CDCl3	9	100
		4.6	95±2
		2.2	35±4
	Acetn	4	95±2
		2.2	64±30
		1	23±2
Propargyl-pyruvate	CDCl3	5.6	100
		2.9	83±5
		1.1	58±2
	Acetn	2	100
		1	88±2
Vinyl-acetate	CDCl3	1.5	100
		0.9	90±5
Propargyl-acetate	CDCl3	1	100
		0.5	85±5
		0.25	35±5

Table ST1: data reported in figure 2.I

Cat%	PPh_3 equivalents	yield
1.1		58±2
0.4%		10±2
1%	1	100
0.6%		98±5
0.4 %		50±3
0.6%	2	100
0.4		98±2%
0.2%		60±5
0.2%	8	100
0.14%		100
0.08%		40±5

Table ST2. Data reported in figure 2.II
